# An evergreen mind and a heart for the colors of fall

**DOI:** 10.1093/jxb/erab162

**Published:** 2021-04-15

**Authors:** Sylvain Aubry, Bastien Christ, Bernhard Kräutler, Enrico Martinoia, Howard Thomas, Cyril Zipfel

**Affiliations:** 1 Department of Plant and Microbial Biology, University of Zürich, Zürich, Switzerland; 2 Berries and Medicinal Plants, Plant Production Systems, Agroscope, Conthey, Switzerland; 3 Institute of Organic Chemistry & Center of Molecular Biosciences, University of Innsbruck, Innsbruck, Austria; 4 Institute of Biological, Environmental and Rural Sciences, Aberystwyth, Wales, UK; 5 Bielefeld University, Germany

**Keywords:** Chlorophyll catabolism, phyllobilin, plastid, senescence, transporter, vacuole

## Abstract

With the finest biochemical and molecular approaches, convincing explorative strategies, and long-term vision, Stefan Hörtensteiner succeeded in elucidating the biochemical pathway responsible for chlorophyll degradation. After having contributed to the identification of key chlorophyll degradation products in the course of the past 25 years, he gradually identified and characterized most of the crucial players in the PAO/phyllobilin degradation pathway of chlorophyll. He was one of the brightest plant biochemists of his generation, and his work opened doors to a better understanding of plant senescence, tetrapyrrole homeostasis, and their complex regulation. He sadly passed away on 5 December 2020, aged 57.

## Teaser

Chlorophyll breakdown is a fundamental process in nature. The elucidation of the involved pathways was the outstanding scientific achievement of the late Stefan Hörtensteiner, to whom this paper is dedicated.

## Introduction

Throughout much of the 20th century, senescence was the concern primarily of crop scientists because of its significance as a yield-limiting factor. For this reason, physiological studies of senescence initially concentrated on photosynthesis and nitrogen partitioning. Plant breeding for improved yield, resilience, and quality incidentally resulted in varieties that were greener for longer (‘stay-green’; Thomas and Ougham, 2014). Until the late 1980s, virtually nothing was known of the mechanism of chlorophyll loss during senescence—in the words of Hendry *et al.* (1987), it was a ‘biological enigma’. Chlorophyll degradation across biomes defines a whole season of the year. During autumn, it sweeps down from the north of the planet in a wave travelling at more than 1 km h^−1^. The progress of fall and the ripening of crops is captured by Earth observation satellites tuned to the wavelengths of the chlorophyll reflectance spectrum (https://go.nasa.gov/2Y1dShZ; Mariën *et al.*, 2019). To have established in molecular detail, down to the level of cells, organelles, enzymes, and genes, the mechanisms underlying this global-scale biological process is a major achievement, for which Stefan Hörtensteiner’s leadership should be celebrated.

**Fig. 1.**
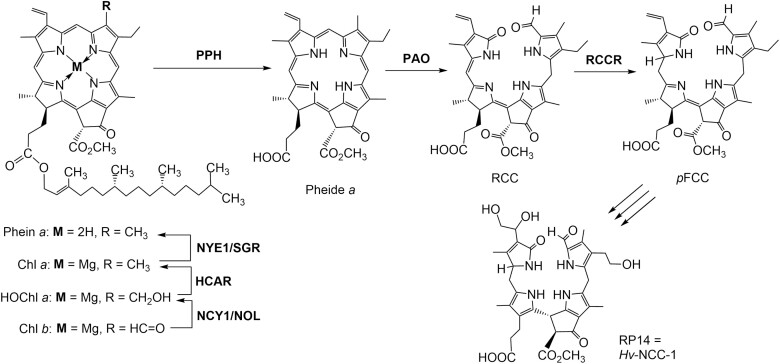
Overview of the first steps of chlorophyll breakdown. The chemical structure of RP14 (=*Hv*-NCC-1), the structures of intermediates (Hörtensteiner *et al.*, 2019), and the enzymes that catalyze each step are shown. NCY1 (NON-YELLOW COLORING1)/NOL, CHLOROPHYLL *b* REDUCTASEs; HCAR, HYDROXYMETHYL CHLOROPHYLL *a* REDUCTASE; NYE1/SGR (STAY GREEN), MAGNESIUM-DECHELATASE; PPH, PHEOPHYTINASE; PAO, PHEOPHORBIDE A OXYGENASE; RCCR, RCC REDUCTASE; Chl *a*, chlorophyll *a*; Chl *b*, chlorophyll *b*; HOChl *a*, hydroxymethyl chlorophyll *a*; Phein *a*, pheophytin *a*; Pheide *a*, pheophorbide *a*; RCC, red chlorophyll catabolite; pFCC, primary fluorescent chlorophyll catabolite.

**Fig. 2.**
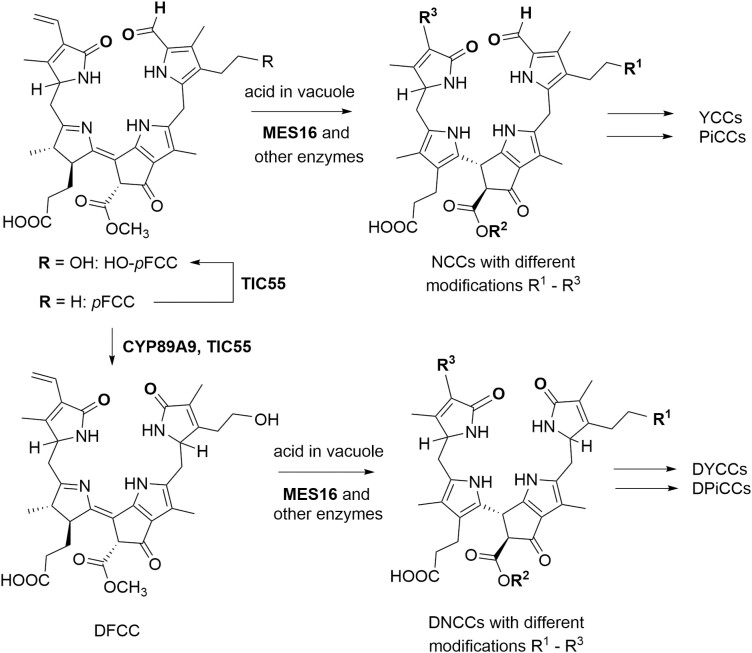
Downstream steps of chlorophyll breakdown in angiosperms. CYP89A9, FCC-DEFORMYLASE; TIC55, TRANSLOCON AT THE INNER CHLOROPLAST ENVELOPE 55; MES16, METHYLESTERASE; *p*FCC, primary fluorescent chlorophyll catabolite; NCC, non-fluorescent chlorophyll catabolite; YCC, yellow chlorophyll catabolite; PiCC, pink chlorophyll catabolite; DFCC, dioxobilin-type fluorescent chlorophyll catabolite; DNCC, dioxobilin-type non-fluorescent chlorophyll catabolite; DYCC, dioxobilin-type yellow chlorophyll catabolite; DPiCC, dioxobilin-type pink chlorophyll catabolite.

The pathway of chlorophyll breakdown, referred to as the PHEOPHORBIDE A OXYGENASE (PAO)/phyllobilin pathway, splits into two: a first chloroplastic section, which leads to the opening of the porphyrin ring of chlorophyll (Fig. 1), and a second section that involves cytosolic and vacuolar processes that produce non-toxic linear tetrapyrroles, phyllobilins (Fig. 2) (Kuai *et al.*, 2017; Hörtensteiner *et al.*, 2019). The PAO/phyllobilin pathway is relatively well described in angiosperms (Figs 1, 2). The first committed step to chlorophyll degradation is the conversion of Chl *b* to Chl *a* by a two-step reaction catalyzed by NONYELLOW COLORING 1 (NYC1) and HYDROXYMETHYL CHLOROPHYLL *a* REDUCTASE (HCAR) (Kusaba *et al.*, 2007; Horie *et al.*, 2009; Sato *et al.*, 2009; Meguro *et al.*, 2011). The pigment is subsequently processed by a magnesium-dechelatase, NON YELLOWING 1 (NYE1) (Shimoda *et al.*, 2016), a dephytylase, PHEOPHYTINASE (PPH) (Schelbert *et al.*, 2009), and PAO, which catalyzes the irreversible opening of the porphyrin ring (pheophorbide *a*) (Pružinská *et al.*, 2003). After these coordinated reactions, which leave no detectable trace of intermediates under normal physiological conditions, a first linear tetrapyrrole is produced, the red chlorophyll catabolite (RCC) (Kräutler *et al.*, 1997). In Arabidopsis, RCC is directly converted to a fluorescent catabolite [the primary fluorescent chlorophyll catabolite (pFCC)] by the enzyme RED CHLOROPHYLL CATABOLITE REDUCTASE (RCCR) (Hörtensteiner *et al.*, 2000; Wüthrich *et al.*, 2000). pFCCs are then modified further and exported from the chloroplast. While the first part of the pathway appears to be relatively straightforward, the fate of pFCCs outside the chloroplast appears to be more diverse and involves various detoxifying enzymes, with more than 40 phyllobilins having been described to date (Hörtensteiner *et al.*, 2019). For example, in Arabidopsis, CYP89A9 and MES16 were shown to catalyze C1 deformylation and O8^4^ demethylation, respectively (Christ *et al.*, 2012, 2013, 2016). With the objective of recognizing Stefan’s major contributions to the understanding of the biochemical processes of chlorophyll breakdown, here we review the process of discovery of the pathway and give a glimpse into future research directions these advances have triggered.

## Identification of the first phyllobilins primed the discovery of catabolic enzymes

The colorless catabolite RP14 from barley is non-fluorescent, and was renamed *Hv*-NCC-1, as it became the first known representative of the abundant non-fluorescent chlorophyll catabolites (NCCs) (Matile *et al.*, 1996). The unambiguous elucidation of the molecular structure of *Hv*-NCC-1 indicated an oxygenolytic cleavage of the macro-ring of chlorophyll (Kräutler *et al.*, 1991). Decisive further momentum was gained by Stefan’s isolation and subsequent determination of the chemical structure of a fleetingly observable fluorescent chlorophyll catabolite (FCC) in senescent leaves of oilseed rape (*Brassica napus*), then named *Bn*-FCC-2, whose chemical structure revealed the nature of the pFCC (Mühlecker *et al.*, 1997). This research provided key molecular insights that allowed Stefan to search for the activity and location of the corresponding hypothetical enzymes (Hörtensteiner, 2006) that catalyze the essential transformations of the chlorophyll catabolites, linear tetrapyrroles that were eventually named phyllobilins (Fig. 1) (Kräutler, 2014).

### PAO/RCCR

Following the identification of the first phyllobilins (*Hv*-NCC-1, *Bn*-FCC-2, etc.), the next logical step was to look for the metabolic process(es) responsible for the porphyrin ring opening. Stefan started this work, initially as a postdoctoral fellow, with some inhibitor and labeling studies, both in angiosperms (Hörtensteiner *et al.*, 1995, 1998) and in algae (Hörtensteiner *et al.*, 2000). In the early 2000s, with the first *Arabidopsis thaliana* genome sequences starting to flow in, cloning of the PAO, a chloroplast-localized Rieske-type mono-oxygenase, was made possible (Fig. 1) (Pružinská *et al.*, 2003). The identification of this key step was one of Stefan’s greatest achievements, carried out while he was an assistant professor at the University of Bern. Indeed, further studies of chlorophyll degradation eventually led to the discovery of an ever-growing list of chemical structures of chlorophyll catabolites (Kräutler, 2016; Hörtensteiner *et al.*, 2019) in the PAO/phyllobilin pathway (Fig. 1) (Kräutler and Hörtensteiner, 2014). Based on the early work, and critically assisted by the structural identification of the first FCCs, the RCC was proposed as a likely intermediate (Mühlecker *et al.*, 1997) and was made available by specific synthesis (Kräutler *et al.*, 1997). This work fostered the identification of the companion enzyme to PAO, namely RCCR, a ferredoxin-dependent bilin reductase (Rodoni *et al.*, 1997), whose corresponding gene was later cloned in Stefan’s group (Pružinská *et al.*, 2005). Interesting work describing the phototoxicity of catabolites accumulating in mutants impaired in these two enzymatic steps brought new insight into the physiological relevance of actively degrading chlorophyll in a tightly controlled way (Pružinská *et al.*, 2005).

### NYE1/SGR

A senescence mutant of the pasture grass *Festuca pratensis* was described by Thomas and Stoddart (1975). The mutant retained chlorophyll while other senescence processes, such as protein breakdown, occurred normally. Grasses of the *Festuca*–*Lolium* complex readily form interspecific and intergeneric hybrids, and this property was exploited to cross the stay-green trait into a range of backgrounds, including a *Lolium perenne* genetic mapping population. The substitution of homeologous alien chromosomal segments facilitated introgression mapping and map-based cloning in these species (King *et al.*, 2007). The recessive *stay-green* mutation transferred from *Festuca* into *Lolium* was located by genomic *in situ* hybridization as a pair of terminal chromosomal segments and, using molecular markers, it was mapped to a sector of *Lolium* chromosome 5. This sector was shown to be syntenic with a region of rice chromosome 9 in which a number of independent studies had identified a major quantitative trait locus for leaf senescence. Fine mapping in *Lolium/Festuca* using common rice markers narrowed down the number of candidate genes to about 30 on a single rice bacterial artificial chromosome. The most likely candidate was *Os09g36200*, a sequence of unknown function homologous to an Arabidopsis senescence-associated gene, *At4g22920*. Knocking out *At4g22920* by RNAi created an Arabidopsis phenotype with all the biochemical features of the original stay-green *Festuca* (Armstead *et al.*, 2006, 2007; Aubry *et al.*, 2008). The gene, now designated *SGR* (also referred to as *NYE1* in Arabidopsis; Ren *et al.*, 2007), is highly conserved across plant species, and further comparative mapping confirmed that allelic variation in it is responsible for the phenotypes of Mendel’s green and yellow pea cotyledons (Thomas *et al.*, 1996; Armstead *et al.*, 2006). The biochemical activity of SGR has been identified only recently, showing that it is the enzyme responsible for the dechelation of the magnesium from the tetrapyrrole ring (Shimoda *et al.*, 2016). Further work involving Stefan’s team has also shown interaction of NYE1/SGR with the photosystems. The functions of SGR/NYE1 and its paralogs (SGR2 and SGRL) are still a matter of debate (Süssenbacher *et al.*, 2019; Xie *et al.*, 2019). Interestingly, an ortholog of SGR in *Chlamydomonas reinhardtii* was shown to be involved in photosystem homeostasis rather than chlorophyll dechelation, data that are consistent with NYE1/SGR interactions with the photosystems in Arabidopsis (Sakuraba *et al.*, 2012).

**Box 1. Key developments: Stefan Hörtensteiner’s career**

After completing his undergraduate study in Würzburg, Germany, Stefan obtained his PhD at the ETH Zürich in the group of Nikolaus Amrhein on vacuolar biogenesis. The earliest successful studies on the catabolism of chlorophyll were carried out by the group of Philippe Matile, at ETH and subsequently at the University of Zürich. Matile had shown that the vacuole served as the plant cell’s lytic compartment, and it was hypothesized that its function during senescence was to facilitate the dismantling of chloroplasts (Wittenbach *et al.*, 1982). A complex picture soon emerged. It was clear that the early events in the degradation of plastid components, including chlorophyll and proteins, took place in the chloroplast itself, and that the vacuole must participate further downstream (Martinoia *et al.*, 1983). A critical discovery was that of a number of putative products of chlorophyll breakdown, informally called ‘rusty pigments’ (RPs) because of the colors they developed when exposed to air (Matile *et al.*, 1987). Subsequently it was shown that RPs accumulate in the vacuole (Matile *et al.*, 1988). An RP from barley (*Hordeum vulgare*), named RP14, isolated in the Matile group by the late Karlheinz Bortlik, was identified unambiguously as a product of the catabolic opening of the chlorophyll macrocycle in work spearheaded by Bernhard Kräutler (Kräutler *et al.*, 1991). Key elements of the chemical structure of this colorless linear tetrapyrrole contrasted with all expectations and provided the insights that allowed for the further productive investigation of chlorophyll breakdown.

Having established the outline of the subcellular organization and metabolic reactions underlying the loss of green color during senescence, the scene was set for a concerted molecular attack on the problem. Stefan took a leading role in this work; he had joined Matile’s lab in 1994 and proceeded to apply all his expertise in biochemistry and molecular biology. As described in this paper, progress was quick, and within 12 years a virtually complete biochemical pathway for chlorophyll breakdown was established by Stefan and his colleagues (Hörtensteiner, 2006), named the PAO/phyllobilin pathway (Kräutler and Hörtensteiner, 2014; Hörtensteiner *et al.*, 2019). Indeed, the major contributions of the different cell structures—plastids, endoplasmic reticulum membranes, cytosol, and vacuoles—had been identified, and most of the genes encoding enzymes and transporters had been cloned and functionally characterized (Ougham *et al.*, 2008).

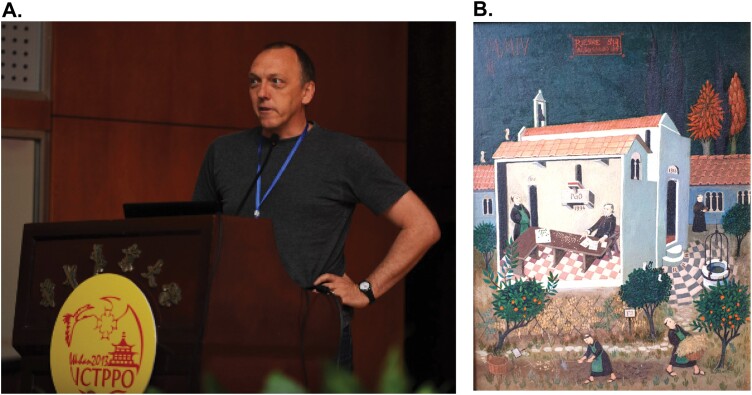

(A) Stefan Hörtensteiner lecturing at the International Conference on Tetrapyrrole Photoreceptors in Photosynthetic Organisms (ICTPPO) in 2013. (Credit: Kai-Hong Zhao). (B) ‘Working on the shoulders of giants’ (painting by the late Prof. Philippe Matile): an imaginative picture summarizing centuries of work in the field of chlorophyll degradation. This work was historically initiated by Gregor Mendel’s work on stay-green peas (in the middle of the picture). Note the cartouche in the upper part representing pheophorbide *a* oxygenase, a Rieske-type mono-oxygenase identified by Stefan Hörtensteiner (SH).

### PPH/TIC55

Stefan, once back in Zürich and promoted to professor, set to work to identify the enzymes catalyzing the formation of chlorophyll catabolites, employing liquid chromatography–mass spectrometry. Two additional chloroplastic enzymes were described, namely PPH and TIC55. PPH is a senescence-specific α/β hydrolase that catalyzes phytol hydrolysis from pheophytin (and not Chl *a*) (Schelbert *et al.*, 2009). Recently, a new potential paralog of PPH has been identified, CLD1, which is thought to be more specifically involved in pigment turnover under stress (Lin *et al.*, 2016). Slightly later, similar creative thinking by Stefan led him to identify a new function for the long-known TIC55 (Hauenstein *et al.*, 2016), initially isolated as a member of the TIC/TOC complex responsible for protein import into the chloroplast (Küchler *et al.*, 2002). *TIC55* was eventually cloned and its protein product was shown to catalyze the hydroxylation, at the C3^2^ position, of pFCC (Hauenstein *et al.*, 2016). TIC55’s function and its derived hydroxylation of phyllobilins is ubiquitous in angiosperms, and the physiological relevance of this hydroxylation is clearly associated with the newly introduced modifications of the new hydroxyl moiety in the downstream phyllobilins (see below).

### MES16/CYP89A9

Using reverse genetics in Arabidopsis, Stefan’s group, in collaboration with the Kräutler group, identified two key enzymes involved in species-specific modification of colorless phyllobilins. MES16, a methylesterase, was shown to demethylate FCCs at the C13^2^-carboxymethyl group present at the isocyclic ring (Christ *et al.*, 2012). Interestingly, this enzymatic activity, which is present only in some plant species, increases the rate of the isomerization of fluorescent phyllobilins in the vacuole, so that senescent leaves of *mes16* null mutants are fluorescent under UV light.

Before the identification of CYP89A9, a cytochrome P450, NCCs were thought to be the major phyllobilins in Arabidopsis. While digging into old high-performance liquid chromatography data, Stefan and his colleagues found out that a *cyp89a9* mutant analyzed several years earlier accumulated up to 10 times more NCCs than wild-type plants. Using genetics and heterologous expression of CYP89A9 in insect cells, they were able to demonstrate that endoplasmic reticulum-localized CYP89A9 catalyzes the oxidative deformylation of a large fraction of some FCCs at C5, generating the corresponding dioxobilin-type FCCs (DFCCs), and is responsible for the subsequent accumulation of (deformylated) dioxobilin-type NCC (DNCCs) in the vacuole (Christ *et al.*, 2013; Hörtensteiner *et al.*, 2019).

## Understanding phyllobilin export from the chloroplast

In the late 1980s the laboratories of Philippe Matile and Howard Thomas had shown that chlorophyll catabolites are localized in the vacuole of senescing barley leaves. This localization could be demonstrated for several breakdown products of the porphyrin ring but not for the released phytol, which is incorporated into plastoglobuli of senescing chloroplasts (Düggelin *et al.*, 1988; Matile *et al.*, 1988; Bortlik *et al.*, 1990). However, it was unclear how the porphyrin catabolites cross the vacuolar membrane. To elucidate this aspect of chlorophyll breakdown, Stefan and his collaborators generated radiolabeled *Bn*-NCC-1 by feeding *B. napus* cotyledons with 4-[^14^C] 5-aminolevulinic acid in the dark. Since it was not possible to isolate sufficient amounts of vacuoles from *B. napus* cotyledons, vacuoles from barley mesophyll cells were used. In an initial experiment, using vacuoplasts and evacuolated protoplasts from *B. napus*, Stefan showed that in this plant, chlorophyll catabolites are also localized mainly in the vacuole. Transport experiments with *Bn*-NCC-1 showed that it had typical characteristics of ABC transporters, since vacuolar uptake of *Bn*-NCC-1 was not affected by the pH gradient but was strictly ATP-dependent and could be inhibited by vanadate (Hinder *et al.*, 1996). The facts that *Bn*-NCC-2 is also transported and that two barley catabolites, *Hv*-FCC-2 and *Hv*-NCC-1, inhibit the transport of *Bn*-NCC-1 suggested that the vacuolar transporter exhibits a broad substrate specificity, a feature often observed for ABC transporters. The fact that FCC may inhibit transport more effectively than the NCCs raised the question of whether FCCs are transported *in vivo* from the cytosol into the vacuole and converted to NCCs within this acidic compartment by an isomerization process (Oberhuber *et al.*, 2003). During this period the first vacuolar ABC transporters were identified, and Stefan took advantage of this progress to start collaborations in order to identify the ABC transporter responsible for the transport of chlorophyll breakdown products into the vacuole. He contributed to two publications showing that the Arabidopsis proteins ABCC1 and ABCC2, as well as ABCC3, are able to transport *Bn*-NCC-1 (Lu *et al.*, 1998; Tommasini *et al.*, 1998). *In vitro* experiments indicated that AtABCC2 was the most active transporter. To verify *in planta* whether this was true, Stefan performed a study in collaboration with Markus Klein, who worked at the same institute. He treated plants of four vacuolar ABCC transporter mutants with 20 ppm ethylene to initiate senescence and analyzed the chlorophyll content of the mutants. He observed that only the *Atabcc2* mutant exhibited delayed chlorophyll breakdown, indicating that AtABCC2 is a major transporter of phyllobilins (Frelet-Barrand *et al.*, 2008). This result is in line with the *in vitro* analysis. The fact that impaired vacuolar transport delays chlorophyll breakdown highlights the importance of the vacuolar transport for this process.

## Phyllobilin diversity and complexity

As became manifest first from the structure of the NCC RP14 (*Hv*-NCC-1), chlorophyll breakdown in senescent leaves generates phyllobilins with remarkable modifications at the peripheral sites of the phyllobilin core (Kräutler *et al.*, 1991). Furthermore, in the course of the past two decades, a variety of phyllobilin structures have been discovered as chlorophyll degradation products from angiosperms, all deriving from the pFCC generated in the chloroplast in the course of the common early steps of the PAO/phyllobilin pathway (Figs 1, 2) (Kräutler, 2016).

The peripheral hydroxyl group introduced into pFCC by TIC55 turned out to be an anchor for attaching a variety of polar sugar or malonate residues. An apparent dihydroxylation reaction by a still unknown enzyme converts the peripheral vinyl into a dihydroxy-ethyl group, as found in *Hv*-NCC-1 (and in other polar phyllobilins). MES16 is responsible for catalyzing the hydrolysis of the methyl ester moiety of pFCC and some of its early products (Christ *et al.*, 2012). Stefan’s group also discovered that the carboxyl acid group liberated by MES16 increases the rate of isomerization of FCCs, in a proposed non-enzymatic, acid-catalyzed reaction, which occurs in the vacuoles of senescent plant cells (Oberhuber *et al.*, 2003). In striking contrast, esterification of the carboxylic acid group of pFCC (Oberhuber *et al.*, 2008) and of its more polar downstream analogues generates persistent FCCs, named hypermodified FCCs (hmFCCs), whose isomerization to NCCs is impaired. Hence, hmFCCs accumulated in the senescent leaves of, for example, banana plants (*Musa acuminata*) (Banala *et al.*, 2010) as well as in ripe banana fruit (Moser *et al.*, 2008a, 2009), causing their remarkable blue glow under UV light—a possible message to frugivores.

Indeed, an entire ‘second line’ of linear tetrapyrroles derived from pFCC became known, resulting from a spectacular deformylation reaction catalyzed by the cytochrome P450 enzyme CYP89A9. This enzyme replaces the characteristic formyl group of its FCC substrate (e.g. pFCC) at the original cleavage site by a second oxo-moiety, producing a corresponding dioxobilin-type FCC (DFCC), the precursors of corresponding dioxobilin-type NCCs (DNCCs) and their further downstream catabolites. As a consequence, two basic types of phyllobilins exist that differ by the nature of the functionality at the site of cleavage of the chlorophyll-derived macroring (Fig. 2), formyloxobilins (e.g. NCCs) and dioxobilins (e.g. DNCCs). Members of both lines of colorless phyllobilins, the NCCs and DNCCs, are substrates for oxidative transformations in senescent leaves that produce brightly colored phyllobilins, collectively named phyllochromobilins. Among these are yellow phyllobilins (YCCs and DYCCs), first described in 2008 (Moser *et al.*, 2008b) and 2019 (Li *et al.*, 2019), respectively, and now classified as phylloxanthobilins, which are readily oxidized further to the corresponding pink products (PiCCs and DPiCCs, classified as phylloroseobilins) (Kräutler, 2016). The phyllochromobilins promise to have interesting biological properties, contrasting with the earlier characterization of the chlorophyll breakdown pathway as a mere detoxification mechanism (Moser and Kräutler, 2019; Karg *et al.*, 2020).

## Outstanding questions: towards understanding the diversity, regulation, and evolution of chlorophyll breakdown

Stefan’s work not only changed our understanding of the chlorophyll degradation process, but also paved the way for future research. In fact, some questions that had been asked in the early 1990s still remain unanswered. For example, the identity and specificity of transport processes that allow the export of (hydroxy-)pFCC from the chloroplast are, despite many years of work, still an open question. As a scientist of his time, Stefan profited from the extensive genetic resources available in Arabidopsis, and most of the biochemical characterization work has been performed in this model species. Today, the rise of phylogenomics, as in many other fields (Young and Gillung, 2020), will surely bring new insight into the diversification of the PAO/phyllobilin pathway during plant evolution, as some data from the Kräutler/Müller laboratories suggest (Erhart *et al.*, 2018). Here, too, early work by Stefan using the green alga *Auxenochlorella protothecoides* showed a route towards identifying possible differences in the ways photosynthetic organisms deal with highly photoreactive chlorophyll catabolites, namely by converting them into more polar compounds to be stored in the vacuole or simply excreting phototoxic intermediates into the surrounding medium (Hörtensteiner *et al.*, 2000).

Now that most of the components of the pathway have been elucidated, much work lies ahead to try to determine the extent to which the PAO/phyllobilin pathway is embedded in gene regulatory networks responsible not only for leaf senescence (Kuai *et al.*, 2017) but for processes such as the hypersensitivity response (Mur *et al.*, 2010). Some of the latest work from Stefan’s laboratory was concentrating on a possible role for pheophorbide *a* as a retrograde signal involved in a feed-forward loop remodeling nuclear gene expression (Aubry *et al.*, 2020). Interestingly, these data suggest that the PAO/phyllobilin pathway might not only be a downstream consequence of the global senescence process, but also participate, in tight association with jasmonic acid signaling, in the regulation of the chloroplast-to-gerontoplast transition. Many other groups also built on Stefan’s work and went further into investigating the hormonal and transcriptional regulation of the pathway (Kuai *et al.*, 2017; Hörtensteiner *et al.*, 2019).

## Conclusion

Despite his very saddening early death, Stefan has contributed a wealth of seminal and fundamental work to the field that will remain much cited. As well as a researcher of the first rank, Stefan was also a keen teacher and mentor, who supervised 12 PhD students, six postdoctoral fellows, and numerous undergraduate students over the years. We will remember Stefan as our colleague, mentor, and friend. While we will try to take over his legacy in pursuing the unravelling of nature’s mysteries, his insightful and passionate comments will surely be missed.
